# Shear Wave Optical Coherence Elastography and Structural Analysis of the Postnatal Mouse Cornea through Development

**DOI:** 10.1002/jbio.202400331

**Published:** 2024-12-02

**Authors:** Andrew L. Lopez, Mohammad Dehshiri, Alexander W. Schill, Salavat Aglyamov, Kirill Larin

**Affiliations:** ^1^ Department of Biomedical Engineering University of Houston Houston Texas USA; ^2^ Department of Mechanical and Aerospace Engineering University of Houston Houston Texas USA; ^3^ Department of Integrative Physiology Baylor College of Medicine Houston Texas USA

**Keywords:** biomechanics, cornea, development, mouse, optical coherence elastography, postnatal

## Abstract

The cornea is a transparent lens at the forefront of the eye, serving as a structural barrier that protects the eye and provides the majority of the eye's refractive power. In this study, we utilized the postnatal mouse eye to characterize corneal structure and biomechanics. Between postnatal day (PN) 6 and PN24, we observed that elastic wave speeds are highest at PN6, gradually decrease through PN24, and then start to rise in adulthood (6 months). We found that corneal thickness is uncoupled from elastic wave speed and that the content and organization of the cornea primarily influence its mechanical properties. To the best of our knowledge, this work is the first detailed assessment of the postnatal mouse cornea's structure and biomechanics and warrants further investigation into the dynamic properties of the postnatal eye.

## Graphical abstract

This paper investigates the geometrical and biomechanical properties of neonatal eyes at the early stages of development. We found that the corneal thickness is uncoupled from elastic wave speed and that the content and organization of the cornea primarily influence its mechanical properties.

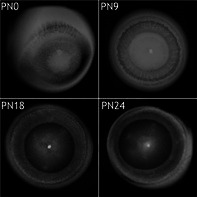

## 1 Introduction

The cornea is a biological optical component that is the primary lens that makes our vision possible. Primarily composed of organized collagen fibers, this thin, translucent structure is a complex network of extracellular proteins, filaments, and fibers inhabited by support cells, mainly keratocytes, that maintain, repair, and remodel the architecture of the lens. As is true with all optical components, the shape of the cornea impacts its function as a lens—to transmit and refract light to focus an image on our retina. The shape of the cornea is formed by an equilibrium between the mechanical properties of the cornea and the intraocular pressure (IOP) of the eye. When this mechanical homeostasis is disrupted, the shape of the cornea is altered, and visual acuity diminishes. For example, the degenerative corneal disease keratoconus, a form of cornea ectasia, leads to a weakening and thinning of the central cornea that causes the cornea to protrude and make a small elevation at the center of the cornea.

In 1970, Hartstein et al. [1] were the first to identify low ocular rigidity as a “new syndrome,” concluding that it positively correlated the use of corneal contact lenses with the development of keratoconus in patients with low ocular rigidity, as measured by a Schiotz mechanical tonometer. Though ocular rigidity was observed as a contributing factor to corneal deformation, surgical interventions and corneal reshaping via contact lenses were mainstays of treatment [2]. In the 1990s, the inception of corneal crosslinking (CXL) directly targeted ocular rigidity by stiffening the cornea and reestablishing mechanical homeostasis [2, 3]. This major advancement in treating corneal disease made corneal stiffness a direct target for measurement and manipulation in the treatment and prevention of corneal disease [4]. With this development, a large focus has been dedicated to developing approaches that measure the mechanical properties of the cornea. This is important for two reasons: first, it provides a measure to diagnose and grade disease, enabling clinicians to design appropriate treatments, and second, it allows clinicians to assess the success of interventions like CXL and monitor a patient's progress [2, 5]. Toward this end, optical coherence elastography (OCE) has emerged as a promising technique to measure the mechanical properties of the cornea [6, 7, 8, 9]. Its high spatial resolution and sensitivity, and ability to be combined with non‐contact excitation sources such as acoustic radiation force [10, 11], make it suitable for clinical and research applications [12]. Additionally, OCE can be a powerful tool for developmental biology where model organisms can be utilized to better understand how biological systems such as the eye form and develop, which has the potential to inform clinical treatments and methods to prevent disease. For our purpose, we have leveraged the advantages of OCE to investigate the development of the postnatal mouse eye to characterize the structural and biomechanical properties of the eye, which has the potential to draw parallels with human eye development since human eyes open at birth but are not fully developed till 2 years of age [13].

**FIGURE 1:**
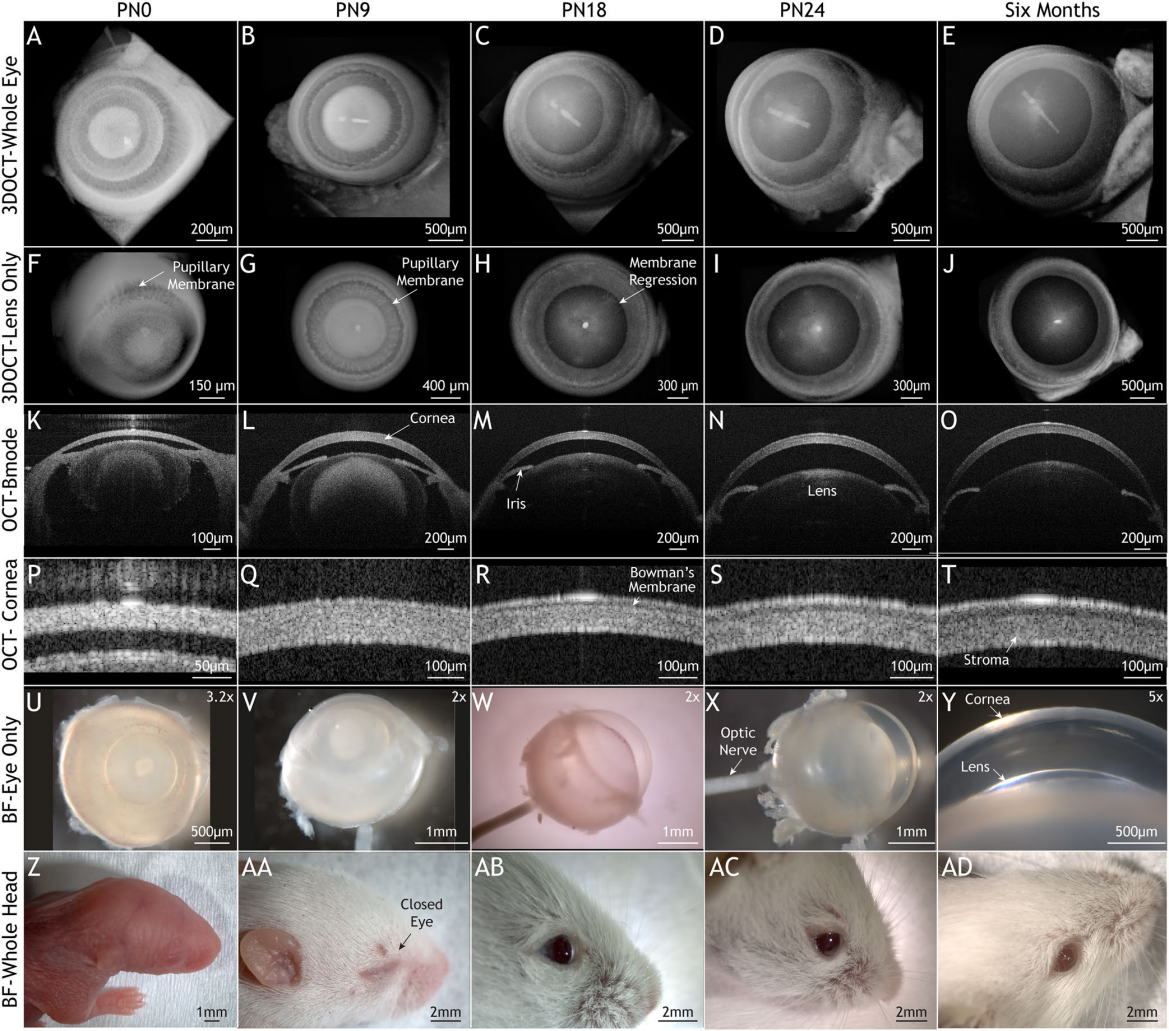
Mouse eye development. Overview of the stages of development in the postnatal eye into adulthood. Panels (A–T) are 2D and 3D OCT images that highlight structural details through mouse development. Panels (U–Y) are bright‐field images of the enucleated mouse eye. Panels (Z–AD) are bright‐field images of the mouse head.

In the developing mouse eye, the primitive cornea is established between embryonic days (E) 12.5–15.5, which corresponds to 6 weeks of development in humans [14, 15]. Briefly, on E12, the early lens detaches from the surface ectoderm, the outermost layer of the embryo, and creates a space that is filled with mesenchymal cells that condense and form layers held loosely by extracellular fibers. Here, the surface ectoderm will become the corneal epithelium, the layer above the lens will become the corneal endothelium, and the layers in between will become the stroma [14]. While the blueprint of the cornea has been established, a great deal of development still takes place in the mouse eye during postnatal development (Figure 1). At birth, the mouse is born with eyes and ears shut, no fur, and completely dependent on their nursing dam for sustenance and warmth (Figure 1Z). The mouse eye opens on approximately PN12 but is not fully functional until PN16 [16]. During this time, the cornea expands and remodels [17], the pupillary membrane of the lens regresses [18, 19], and the lens becomes transparent by the rearrangement and compaction of collagenous fibers [20] (Figure 1A–J). After PN24, the postnatal eye continues to grow and remodel but at a slower rate that will level off into early adulthood, when the biological program switches from development to homeostasis.

**FIGURE 2:**
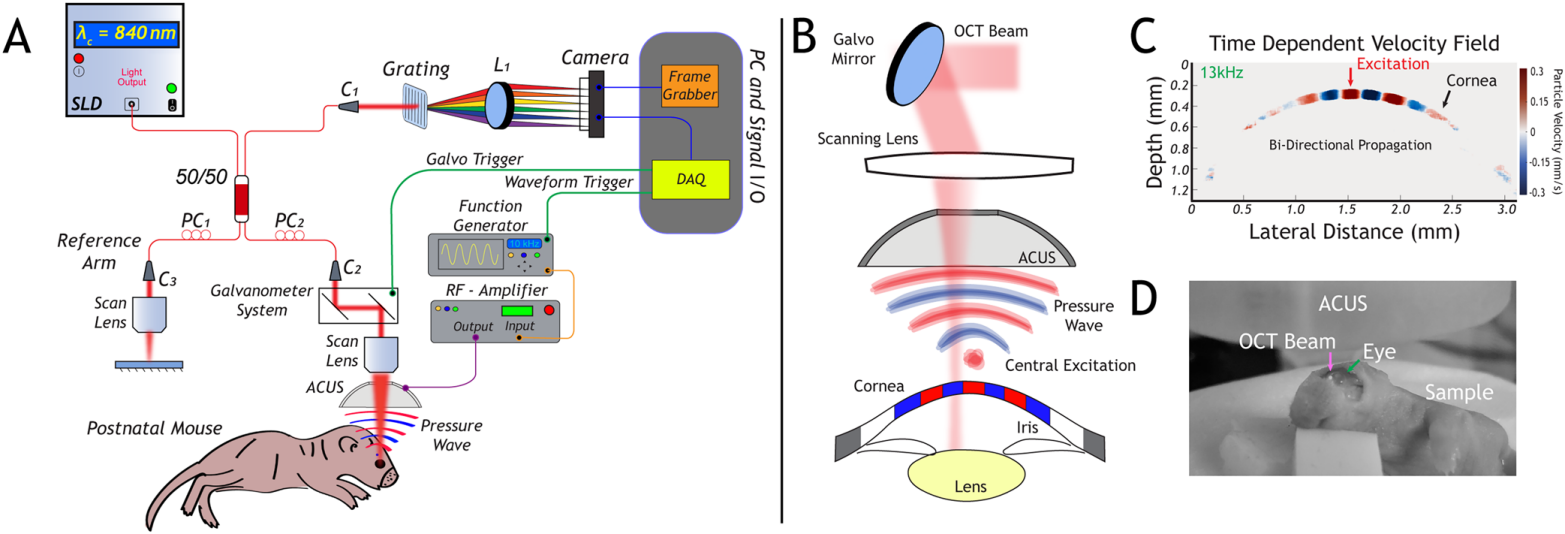
OCE system and technique. Panel (A) is a schematic of our lab‐built OCE system based on a phase‐sensitive SD‐OCT system. (C) (1–3): collimator. PC (1, 2): polarization controllers. L1: cylindrical lens. Panel (B) illustrates the coaxial registration between the ACUS and OCT scanning lens. The ACUS is focused on the apex of the cornea for central excitation and generates a bi‐directional mechanical wave, as illustrated in panel (B) and shown in panel (C). Panel (D) is the sample mounted on the imaging stage.

While many of the cellular, molecular, and genetic mechanisms have been assessed during these processes, the biomechanical properties of the developing postnatal mouse eye have not been assessed. Furthermore, accurate structural assessments of the postnatal mouse eye are lacking due to its small fragile nature and the variety of tissue processing methods that poorly preserve tissue structure [21]. To the best of our knowledge, in this work, we present for the first time a characterization of the biomechanical properties of the postnatal mouse eye through postnatal development, as the cornea remodels and goes through significant morphological changes. Utilizing optical coherence tomography (OCT) as an imaging modality, we imaged eyes without the need for any labeling to obtain high‐resolution images of the eye in cross‐section (Figure 1K–O). Using custom algorithms, we assessed central cornea thickness (CCT) and the anterior and posterior ROC throughout development (Figure 4). We identified that CCT is dynamic, whereby the cornea's CCT increases rapidly to a maximum at PN12 and then begins to thin until PN18 when the CCT begins to rise again and level off as the mouse enters early adulthood. Using OCE, we co‐register an air‐coupled ultrasound (ACUS) transducer to the OCT sample arm to excite and image elastic wave propagation (Figure 2). We observed that elastic wave speed does not correlate with cornea thickness. On PN6, we measured the highest elastic wave speeds and observed that elastic wave speeds continuously decline through development until adulthood (Figure 5A,B). From our OCT analysis, we have determined that the cornea is a dynamic tissue that undergoes significant remodeling, as shown with non‐linear CCT values between PN6 and PN24 (Figure 4A), and the appearance of new structures such as Bowman's membrane (Figures 1R–T and 2D). Previous work has corroborated our findings, showing that the postnatal mouse cornea swells from PN0 to a maximum on PN12, and is attributed to a high cellular density of keratocytes, which are in the process of moderating cornea remodeling [22]. Thereafter, these cells enter a phase of apoptosis and concurrently the cornea begins to thin until additional layers of collagen fibers are deposited to strengthen and reinforce the cornea to make it stable for the rest of the animal's life.

**FIGURE 3:**
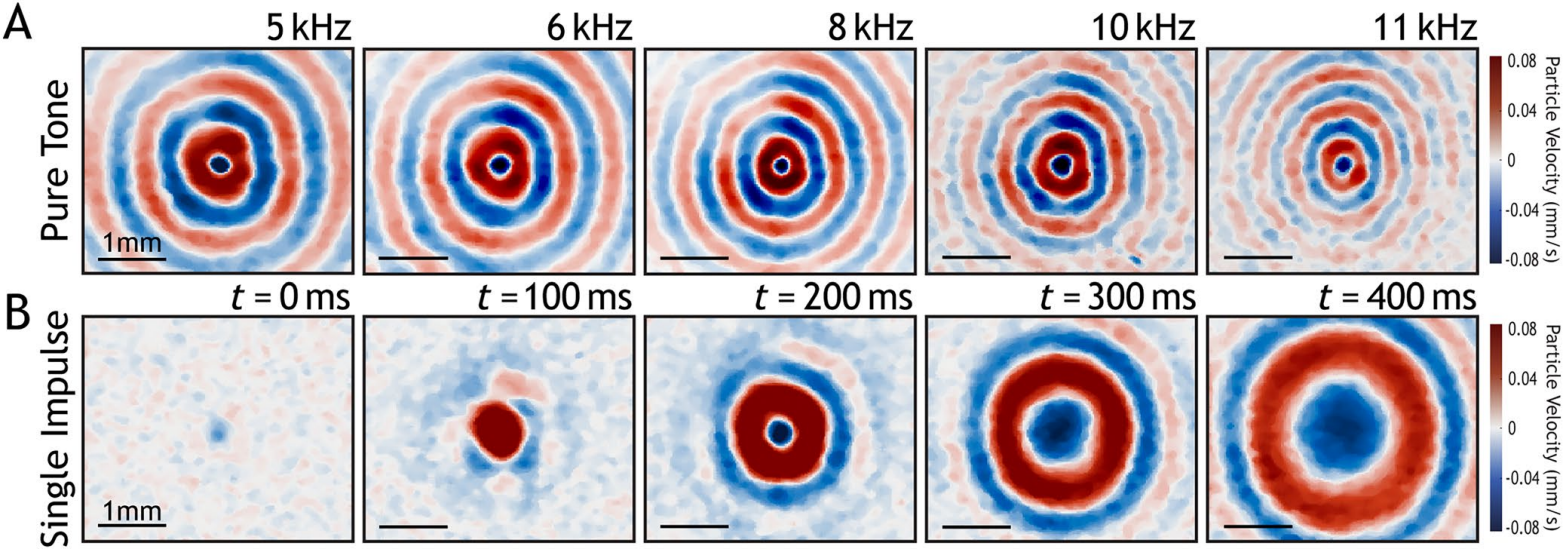
Time‐dependent velocity field of agarose phantom for different waveforms. Panel (A) depicts motion snapshots of pure tone excitation at 5, 6, 8, 10, and 11 kHz. Panel (B) is several selected time frames from a 50 μs singular square pulse.

Taken together, our observation that elastic wave speeds do not correlate with corneal thickness suggests that the mechanical properties of the developing postnatal mouse cornea are dependent on biological mechanisms of development that regulate the content and organization of the cornea. Delineating the mechanisms that regulate the stiffness of the postnatal mouse cornea will provide a clearer understanding of the processes that make a normal, functional cornea.

## 2 Materials and Methods

### 2.1 Mouse and Postnatal Pup Manipulations

All animal manipulation procedures have been approved by the Institutional Animal Care and Use Committee at the University of Houston. Mating cages between CD‐1 mice were set, and mice were checked daily for vaginal plugs. After observing a plugged female, the female mouse was separated and placed in a separate cage with another female to prevent single housing and isolation. The mouse gestational period is approximately 21 days, therefore, at 3 weeks, the pregnant female was observed daily to record an accurate date of birth. The date of birth was recorded as postnatal day 0.

From our initial studies, we observed that the postnatal eye is sensitive and fragile. For example, if a postnatal pup is euthanized and the eye is exposed for an extended period (approximately 10 mins), we observed that the cornea would begin to thin and the space between the cornea and the lens begins to collapse. This was especially true for the early‐stage postnatal pups from PN0 to PN6. To overcome this challenge, we did not use postnatal pups younger than PN6 for OCE experiments. We observed that mechanical excitation of the cornea would induce damage in the lens and flatten the cornea in early postnatal pups. For stages PN6 and higher, a whole litter of pups (8–12 animals) was used for each experiment, and only one eye from one pup was used for each OCE acquisition. We alternated between left and right eyes after each acquisition.

To prepare a mouse pup for OCE imaging, our workflow is as follows. One mouse pup was removed from its cage, while the other littermates were left with their nursing dam to keep normal conditions and physiology. Before the beginning of the procedure, the pup was placed in an empty cage with bedding on top of a heating pad maintained at 37°C, to keep the pup in the most normal conditions possible. Immediately after euthanasia, under a dissection microscope, #5 forceps were used to remove the eyelids (Figure 2D). Before imaging, the postnatal mouse eye was rinsed with 1 × PBS, pH 7.4 to wash away any debris and maintain moisture. The mouse pup was then mounted onto the imaging stage, and with the use of an x‐y translation and goniometric stage, the eye was centered and leveled in the OCT imaging plane. The focus was brought to the apex of the eye and was determined when the autocorrelation noise saturated the signal. To improve SNR, we obtained images with a small offset from the apex to reduce autocorrelation noise.

**FIGURE 4:**
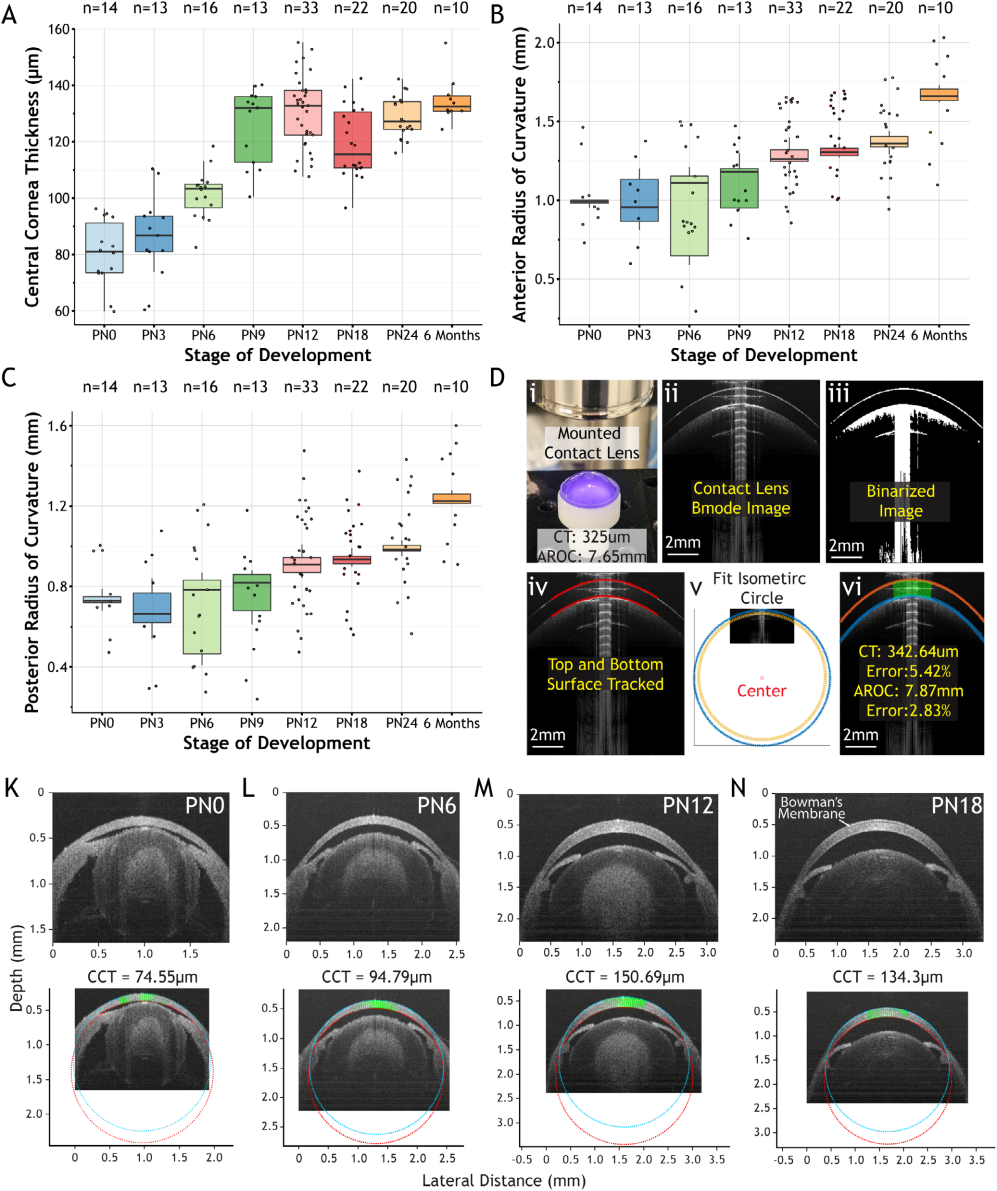
Mouse postnatal eye central cornea thickness and radius of curvature. Panels (A–C) are boxplots that summarize CCT, anterior and posterior radii of curvature, respectively. Panel (D) illustrates our algorithm used to measure CCT and ROC. Panels (K–N) are B‐mode OCT images of postnatal mouse eyes at different stages of development. The second row of panels (K–N) shows an isometric circle fitted to the top and bottom surface of the cornea.

For one OCE experiment, the mice were divided into two groups. In the first group, a single 50 μs pulse was used to measure the group velocity of the broadband propagating elastic wave. The single pulse excitation is also called transient excitation. In the second group, excitation was performed using multiple pure tones using a train of 5 pulses to enable propagation of the quasi‐harmonic elastic wave. In order to make a dispersion curve, the quasi‐harmonic excitation was performed at 5, 6, 8, 10, and 11 kHz five‐cycle pulse trains. Following the OCE measurement, 3D OCT scans were performed for structural imaging of the mouse corneas.

### 2.2 OCE System and Validation

As illustrated in Figure 2A, we utilize a custom‐designed OCE system that incorporates an external excitation apparatus to induce elastic waves in the mouse cornea. To visualize and measure the propagation of elastic waves, a phase‐sensitive spectral domain OCT (SD‐OCT) system is utilized in the configuration. The phase‐sensitive SD‐OCT system is based on a superluminescent diode (Superlum Diodes Ltd., Carrigtwohill, Co. Cork, Ireland) with a central wavelength of 840 nm and bandwidth of 50 nm, which provides an axial and lateral resolution of 9 and 11 μm (in air), respectively, and a displacement sensitivity of 0.28 nm. For the excitation, an ACUS‐OCE probe, which consists of a hollow hemispherical piezo ceramic disc, was utilized. The ACUS is coaxially aligned with the OCT objective lens using a 3D printed holder [11]. The transducer has a focal distance of 20 mm and a resonant frequency of 1 MHz. To implement a non‐contact transient and quasi‐harmonic excitation with the ACUS‐OCE probe, the continuous signal of the resonant frequency at 1 MHz was modulated with a 50 μs square pulse and quasi‐harmonic modulation from 5 to 11 kHz, respectively. An excitation signal was generated using a function generator (DG4162, RIGOL Tech, Beijing, China) and amplified with a radio‐frequency amplifier (1040 L, Electronics & Innovation Ltd., Rochester, NY, USA). To capture wave propagation in the mouse cornea, an M‐B mode scanning protocol was performed, which consisted of 1000 A‐line repetitions (10 ms) at each measurement position out of the total 500 lateral positions. A complete M‐B mode acquisition lasted 5 s. Using this protocol, dispersion curves were generated by exciting quasi‐harmonic excitation waves at 5, 6, 8, 10, and 11 kHz. Transient excitation with a single pulse with approximately a pulse duration of 50 μs was used to determine group velocity. Our initial studies determined that longer wavelengths at excitation frequencies of 2 and 3 kHz were not suitable for the postnatal mouse eye for transient or quasi‐harmonic excitation because of eye's small dimensions and thin cornea. We decided on a 50 μs pulse for transient excitation because of the smaller spot size that it generates and the visible propagation in the postnatal cornea. Additionally, we settled on quasi‐harmonic excitation waves at 5, 6, 8, 10, and 11 kHz because they were the lowest frequencies that would interact with the postnatal cornea. To validate our OCE excitation protocol, we utilized agarose tissue‐mimicking phantoms. The phantom is prepared by mixing 1 × PBS, pH 7.4 with low‐melting agarose powder to a concentration of 0.5% (m/v). The mixture is gently boiled and stirred with a magnetic bar on a hot plate. After assuring that the agarose is dissolved, the solution is allowed to cool and then poured into several 10 cm petri dishes and cured at 4°C. Figure 3 illustrates the enface projected time‐dependent velocity field on an agarose tissue‐mimicking phantom. In Figure 3A, it is clear that as frequency is increased, attenuation increases, and wavelength decreases. In Figure 3B, we can observe the sub‐millimeter spot size at *t* = 0 and the propagation of the transient wave over several millimeters.

**FIGURE 5:**
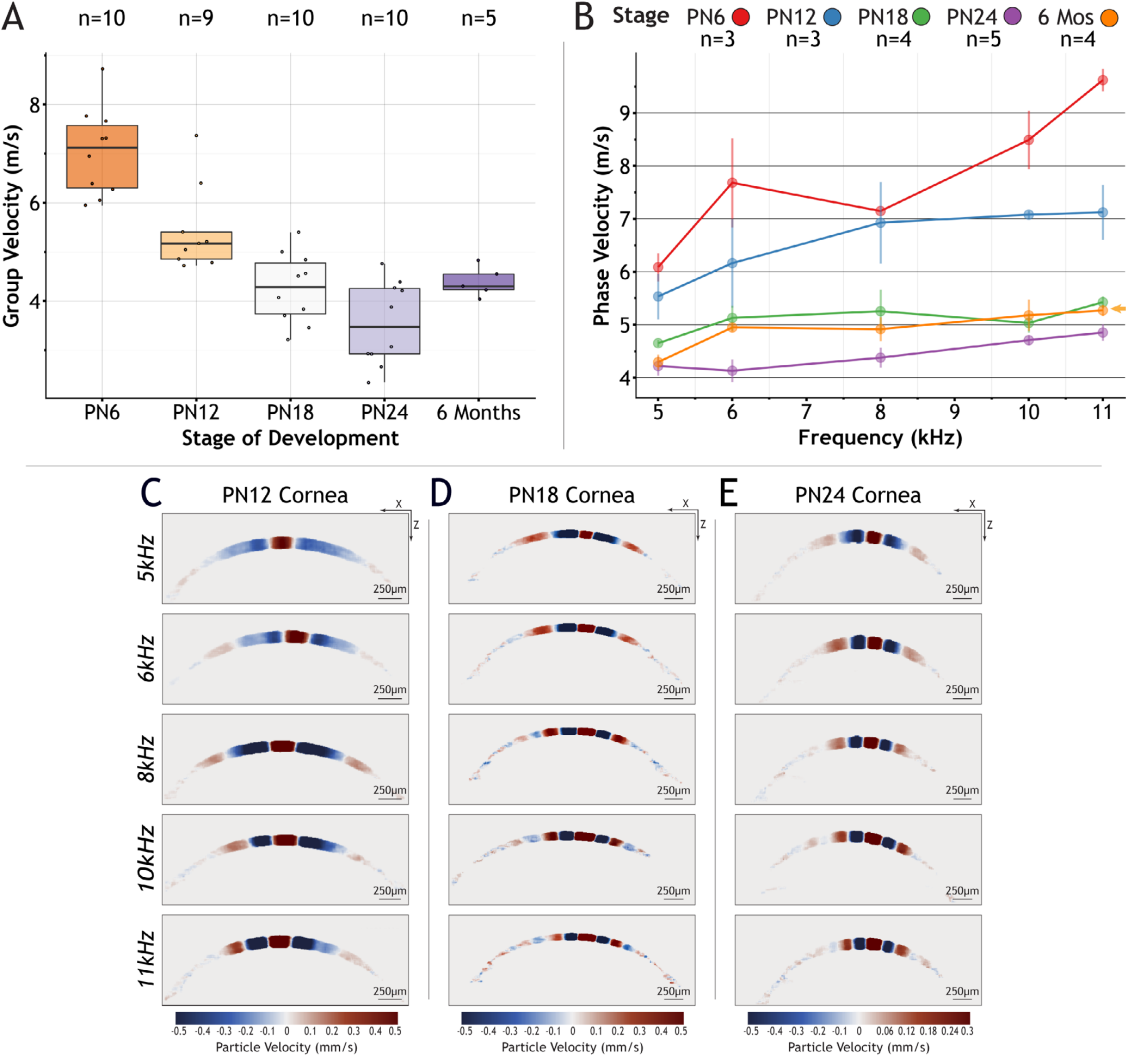
Elastic wave measurements. Panel (A) is a boxplot that summarizes group velocity values obtained at different stages of development. Panel (B) shows dispersion curves generated by exciting pure tones at different stages of development. Panels (C–E) are a collection of particle velocity motion snapshots that show the change in wavelength is dependent on the stage of development at a constant frequency.

### 2.3 Data Acquisition and Processing

Data processing and analysis were performed using a custom MATLAB R2023a (Mathworks Inc., Natick, MA, USA) program. Elastic wave velocity, CCT, and anterior and posterior ROC were measured for each group of pups across developmental time points. To measure the wave speed at the different quasi‐harmonic frequencies and for the transient case, the OCT phase data were processed, and the z‐projection of the displacement was used to extract the elastic wave propagation within the tissue. Then, acquired data were reorganized into a 2D + Time format to visualize the slope of the particle velocity. Implementing a simple bounded search algorithm for maximum values, the main elastic wave component can be tracked. Using linear regression analysis, the slope of the points of maximum amplitude provides a wave speed estimate.

To determine the CCT and ROC, the B‐mode image of the postnatal mouse eye was made into an isometric image using two‐dimensional interpolation. The image was then thresholded and binarized. A custom algorithm was used to zero‐selected regions of the image. This was necessary when threshold binarization failed to distinguish clear boundaries, which often occurred with autocorrelation noise that is hard to avoid when focused on the apex of the eye. A custom algorithm was used to track the top and bottom surfaces of the cornea in the binarized image. The tracking starts and ends at a user‐defined location in the image, which in our case was defined as the location where the cornea and iris bifurcate at the limbus. The positions of the pixels are then used to fit a circle that will define the anterior and posterior ROC for the cornea. CCT was measured by recording the max z‐position of the anterior and posterior circles, taking the difference between z‐positions of the corresponding circles at 10 pixels to the right and 10 pixels to the left of the max z‐position. These values were summed, averaged, and divided by the refractive index of 1.41 [21]. Figure 3D, illustrates our workflow and validation of our measurements made on a rigid contact lens with a measured thickness of 325 μm, an anterior ROC of 7.65 mm, and a refractive index of 1.42. The contact lens was manufactured in‐house and cut from Boston XO material with a DAC lathe (DAC International Inc. Carpinteria, CA, USA).

## 3 Results

### 3.1 The Transient Developing Mouse Eye

The mouse is born blind and nude with its eyes and ears shut (Figure 1Z–AA). Before and after eye opening on PN12, the postnatal eye undergoes a significant amount of morphological and structural changes that are necessary for the proper formation and function of the eye. In Figure 1A–J, volumetric OCT scans reveal the transient structures of the developing eye. The 3D OCT scans of the whole eye and lens show the presence of the pupillary membrane—a transient capillary bed that supports the eye—at postnatal stages 0, 9, and 18 and capture their regression through later stages. The regression of the pupillary membrane is a required step in human and rodent development to allow light to pass through the eye unimpeded, and when this process fails, for example, in persistent pupillary membrane (PPM), vision deficiencies may result [19]. In Figure 1K–O, OCT B‐mode images show the eye in cross‐section. At PN0, the cornea is relatively flat, the anterior chamber is small, the iris is not remodeled, and the lens is opaque. As development proceeds from PN0 to PN24, we can observe significant morphological changes in the eye, such as the increase in corneal curvature, expansion of the eye, increased transparency of the lens, and the remodeling and arrangement of the iris. In Figure 1K–O, we observe the growth and development of the cornea in detail. Figure 1Q, shows the important phase of corneal swelling at PN9, which starts at PN6 and reaches a maximum by PN12. Figure 4A, quantifies this rapid growth and shows the largest difference between developmental stages is from PN6 to PN9 with a change in CCT thickness of approximately 25 μm. Figure 4K–N, illustrates the dynamic growth of the cornea with our method of measurement by applying isometric circles to the OCT B‐mode image to measure CCT and ROC. This process of corneal swelling is attributed to a high cellular density of keratocytes and increased spacing between collagen fibers [20, 22]. After PN12, the cornea enters a phase of cell apoptosis and collagen fiber rearrangement that leads to corneal thinning [13], as shown in Figures 1R and 4N. Figure 4A, shows that CCT thickness is a dynamic non‐linear process that fluctuates as a consequence of developmental processes that regulate the formation of the cornea.

In Figure 4B,C, the anterior and posterior ROC of the postnatal eye are summarized. We observed an initial drop in the average anterior and posterior ROC from PN0 to PN3 and propose that this is due to PN0 corneas being flatter compared to later stages, which leads to a higher ROC measurement (Figure 4B,C). Throughout the rest of postnatal development, ROC continuously increases until it reaches a stable value in adulthood of approximately 1.65 mm for anterior ROC and 1.22 mm for posterior ROC.

In addition to the dynamic changes in CCT and linear growth in ROC, we also observed the appearance Bowman's membrane—an acellular thin region that is situated between the corneal epithelium and stroma—that coincides with the point of maximum thinning at PN18 in Figures 1R and 4N. While a more detailed analysis may reveal that Bowman's membrane appears slightly earlier than PN18, our observation shows that the formation of Bowman's membrane occurs during the process of corneal thinning between PN12 and PN18. Our data suggest that corneal thinning is a part of the remodeling process that leads to the formation of Bowman's membrane.

In Figure 1U–Y, bright‐field stereoscope images complement our OCT images and reveal that the early eye is more oblate rather than spherical (Figure 1U–W). Figure 1U,V, show the oblate enucleated eye with the vasculature of the pupillary membrane covering most of the eye's surface, in contrast, later stages show the eye becomes more spherical, increases lens transparency, and expands its anterior chamber to allow the cornea to take its proper shape.

Finally, in Figure 4D, we illustrate our validation of CCT and ROC in the postnatal mouse eye by applying our algorithms to an in‐house manufactured rigid contact lens. Figure 4Di, shows the rigid contact lens mounted under the scanning lens of our OCT system. Figure 4Dii–v, shows our image processing steps. Briefly, we binarize the OCT B‐mode image and track the top and bottom surfaces of the contact lens. We use the values obtained from the tracking algorithm to fit an isometric circle and measure the anterior and posterior ROC. As shown in Figure 4K,L, the green lines represent the distance between the pixels of the two circles used to measure the anterior and posterior ROC. These distances are summed, averaged, and divided by the refractive index of the material to obtain a measurement of thickness.

### 3.2 Postnatal Mouse Eye Biomechanics

To examine the biomechanical properties of the postnatal mouse eye, we utilized a custom‐designed OCE system that incorporates an external excitation system that is coaxially aligned with the OCT scanning lens to induce an elastic wave on the postnatal cornea (Figure 2A,B). In this arrangement, as shown in Figure 2D, we position the postnatal mouse directly beneath the ACUS to excite an elastic wave at the center of the cornea and generate a bi‐directional wave that propagates away from the center of the cornea (Figure 2C).

As shown in Figure 4A, the average CCT of the postnatal mouse ranges from approximately 80 μm at PN0 to 128 μm at PN24 and reaches an average maximum of 134 μm in adulthood. For these values of CCT, we found that a frequency range of 5–11 kHz was optimal for corneal elastography. We settled on a 10 kHz (50 μs) single impulse to measure group velocity in the postnatal cornea. Longer duration pulses would not induce a wave with a measurable propagation. Also, our initial attempts at lower pure tone frequencies showed that longer waveforms would not interact with the cornea. In Figure 3A,B we tested our OCE protocol in agarose phantoms and demonstrated that our approach generates reproducible shear waves. Finally, we excluded PN0 and PN3 because corneas at this stage were exceptionally fragile and difficult to measure.

In Figure 5A, we observed group velocity values decrease from PN6 to PN24, followed by an increase in adulthood. Striking from this result is the observation that group velocities dropped as corneal thickness increased between PN6 to PN12. It is known that the speed of guided Lamb waves propagating in the cornea is proportional to corneal thickness [23, 24, 25]. However, based on our results, the developing postnatal mouse eye is dynamic and undergoing morphological and structural changes. As a consequence, the relationship between corneal thickness and wave speed is uncoupled. Following this observation, we generated dispersion curves from pure tone excitations. Figure 5B, shows phase velocity color coded by stage of development. We observed that PN6 has the highest velocities compared to later stages, which is in agreement with our group velocity data. The phase velocities measured at PN6 showed a higher variance compared to later stages. We believe this is due to PN6 corneas being more susceptible to damage induced by the ultrasound transducer. For example, in the pure tone experiments, the same eye is imaged several times as frequency is increased and PN6 eyes are more sensitive to repeated excitation. 3D OCT scans acquired after the OCE acquisition did not reveal visible damage in PN6 mice, but excessive force must be avoided in early‐stage postnatal mouse eyes.

Later stages showed a more standard phase velocity profile where values begin to asymptote at higher frequencies. Also, we have observed that dispersion curves for adult mice (6 months) start to increase after PN24. The adult mouse dispersion curve is highlighted with an orange arrow in Figure 5B. The observation that adult mice have increased elastic wave velocities aligns with the fact that young adult mice have corneas that continue to stratify and become reinforced up to 8 weeks after birth [13]. Figure 5C–E, are motion snapshots from selected stages of development. Within a stage of development, we observe that the wavelength of the propagating wave decreases as the frequency of excitation is increased. However, when we compare across stages of development, we observe that the wavelength shortens while maintaining a constant frequency. For example, Figure 5C, shows a PN12 cornea excited at 5 kHz has a longer wavelength compared to PN24 cornea in Figure 5E at 5 kHz. This pattern is consistent across frequencies at later stages of development shown in Figure 5C–E. Our conclusion from these data is that the dynamic processes that occur during postnatal mouse corneal development uncouples thickness from wave velocity, and that the content and organization of the cornea are the primary regulators of the biomechanical properties of the cornea.

## 4 Discussion and Conclusions

The purpose of this study was to investigate the biomechanical properties and structural changes in the developing postnatal mouse eye. To the best of our knowledge, an assessment of the postnatal mouse eye's biomechanical properties has not been completed at these early stages. Furthermore, we have provided a detailed assessment of the postnatal mouse eye's CCT and ROC, and addressed a need for more accurate measurements of the postnatal mouse eye's geometry, which was lacking [21].

In this work, we observed that corneal thickness is uncoupled from elastic wave velocity during postnatal mouse development. While this observation does not follow the trend that the speed of guided Lamb waves propagating in the cornea is proportional to corneal thickness [23, 24, 25], we assert that the dynamic, morphological changes that are occurring in the postnatal eye lead to a softening of the cornea from PN6 to PN24. During these stages of development, the cornea is under dynamic processes that are revealed by our observation that CCT fluctuates through development with an immediate increase in CCT from PN6 to PN12, followed by a thinning that reaches a minimum at PN18 (Figure 4A). Additionally, we observed that between PN12 and PN18, Bowman's membrane appeared as a new structure and continued to expand from PN18 to adulthood. Taken together, these data suggest that the content and organization of the eye are not equal among different stages of development and that the observed elastic wave velocities are indicative of the biological changes occurring in the cornea.

Previous studies have shown that the postnatal mouse cornea fluctuates in thickness. Sheppard et al. [20] assessed corneal fiber alignment through postnatal mouse development and concluded that the postnatal mouse cornea reaches a peak thickness by PN12 due to increased interfibrillar spacing measured via X‐ray scattering. After PN12, the spacing between fibers decreases until it reaches a minimum spacing at approximately PN20 when the cornea begins to expand again. Song et al. [22] analyzed cornea thickness in postnatal mouse eyes with confocal microscopy. They observed corneal swelling that peaked on PN12 and then corneal thinning that reached a minimum at approximately PN18. Using confocal microscopy, this group measured keratocyte cell density to be the highest at PN10 and measured its gradual decrease where most keratocytes had sloughed off by PN20. Furthermore, they confirmed their findings by analyzing mice null for lumican, an essential extracellular matrix protein, and observed that these mice did not experience corneal swelling, maintained a higher level of corneal opacity, and sustained higher levels of keratocyte density. While the eyes of lumican‐deficient mice did become thinner after PN12, the phenotypic outcomes indicated that corneal swelling is necessary for the cornea's preparation to function as an optical lens. Both studies describe a mechanism of corneal swelling that marks a process of preparation for the cornea to become transparent and enable vision. In our work, we have observed corneal swelling and have shown that corneal stiffness does not correlate with corneal thickness. It is apparent that developmental processes are taking place within the cornea in preparation to become a functional lens and facilitate vision. In our assessment, these processes uncouple corneal thickness from elastic wave speed.

In our work, we initially attempted to conduct OCE on PN0 and PN3 mice. While we were successful at exciting single impulse waves in PN3 mice, it became challenging to generate dispersion curves for these mice and at earlier stages. Earlier stages generated velocities and elasticity values that were too high to be considered part of this study. Part of the challenge is exciting a mechanical wave that will properly propagate on an eye that has a cornea that is approximately 80 μm thick and has less than 500 μm to propagate (Figure 1K). Normally, OCE is done in human or porcine eyes, which are closer to 500 μm thick and are large enough to allow wave propagation for several millimeters [8, 26]. For future studies, the use of ultrasound transducers with a higher resonant frequency, the use of piezo‐driven acoustic radiation force transducers, the use of advanced OCE approaches such as reverberant OCE [27], and/or the use of these technologies in conjunction with optical coherence microscopy provide an opportunity to tackle the challenge of imaging early postnatal eyes that are currently too small to properly assess.

One limitation of this study was the inability to control IOP in postnatal mice. While our group has established protocols for controlling IOP when measuring the mechanical properties of the cornea in larger animal models such as the porcine eye [11], controlling IOP in the postnatal mouse eye presents several challenges. First, at early stages, the postnatal eye is fragile, and unlike more mature eyes, they don't have reinforced corneas with extra layers of collagen. From our experience, the postnatal mouse eye will begin to degrade if exposed to the environment for a duration longer than 10 min. Additionally, the use of inflation to control IOP is considered a destructive method and is not suitable for postnatal eyes [28]. Therefore, to ensure each OCE acquisition was of a normal, undamaged eye, we preferred to image immediately after euthanasia. Furthermore, the eye at different stages of development will have different normal IOPs, and while there is a need to determine these values for future experiments, this was beyond the scope of this work. Second, while veterinary tonometers are available, there are no commercially available tonometers capable of measuring IOP in the postnatal mouse eye. To gain a better understanding of postnatal mouse eye biomechanics it will be necessary to determine how IOP changes through development and then design a custom system to accurately maintain normal IOP when examining the postnatal mouse eye.

In this study, we have asserted that the postnatal mouse cornea is dynamic and regulated by biological processes that change the composition and organization of the cornea. Consequently, the thickness of the cornea at postnatal stages does not correlate with elastic wave velocity. However, previous studies have concluded that the radius of curvature is a significant factor that influences wave velocity in tissue‐mimicking phantoms and finite element analysis [24]. While in this study, we have measured the radius of curvature and observed that the postnatal mouse ROC increases from approximately 1 mm at PN0 and increases to approximately 1.65 in adulthood, the ratio between thickness and radius of curvature at each stage of development is less than 10%, which at these small ratios have shown that curvature has small effects on propagation speed [28].

Finally, in this work, we have analyzed the structural formation of the postnatal mouse eye and measured the elastic shear wave speed across the cornea as a function of development. Our conclusion is that the postnatal mouse cornea is a dynamic structure that is continuously remodeling as the mouse eye develops. Because of this, we have revealed the elastic wave speed is uncoupled from corneal thickness. The normal convention is that wave velocity correlates with increased corneal thickness. However, from our results, we have observed that elastic wave speed was the highest at PN6 when the cornea is relatively thin, and that those speeds continue to drop while the cornea experiences a phase of corneal swelling that peaks at PN12 and then a phase of corneal thinning that reaches a minimum at PN18. Elastic wave speeds start to climb in mouse adulthood when the cornea is stable and layers of collagen are deposited to reinforce and maintain the cornea's strength and structure.

Looking forward, future analysis of the postnatal mouse eye will require a multi‐modal approach to better probe the corneal dynamics and delineate why corneal thickness becomes uncoupled from elastic wave speeds. As a next step, the use of optical coherence microscopy and non‐linear imaging modalities such as second harmonic generation (SHG) imaging would provide better insight into the fiber content and organization of the cornea. Furthermore, since these modalities do not require stains for contrast, they open the possibility to live imaging, where it would be possible to assess the dynamics of corneal formation. Having a deeper understanding of how the cornea forms will be useful in designing simulations and experiments to delineate the biomechanics of the postnatal mouse eye.

## Conflicts of Interest

KVL has a financial interest in Elast Eye LLC, which is unrelated to this work.

## Data Availability

The data that support the findings of this study are available from the corresponding author upon reasonable request.
